# Prediction of herbal medicines based on immune cell infiltration and immune- and ferroptosis-related gene expression levels to treat valvular atrial fibrillation

**DOI:** 10.3389/fgene.2022.886860

**Published:** 2022-09-28

**Authors:** Feng Jiang, Weiwei Zhang, Hongdan Lu, Meiling Tan, Zhicong Zeng, Yinzhi Song, Xiao Ke, Fengxia Lin

**Affiliations:** ^1^ Cardiology Department, Affiliated Baoan TCM Hospital, Guangzhou University of Traditional Chinese Medicine, Shenzhen, China; ^2^ Wenhua Community Health Service Center, Shenzhen Luohu Hospital Group, Shenzhen, China; ^3^ Department of Cardiology, Fuwai Hospital, Chinese Academy of Medical Sciences, Shenzhen(Shenzhen Sun Yat-sen Cardiovascular Hospital), Shenzhen, China

**Keywords:** immune cell infiltration, ferroptosis, atrial fibrillation, herbal medicine, prediction

## Abstract

Inflammatory immune response is apparently one of the determinants of progressive exacerbation of valvular atrial fibrillation(VAF). Ferroptosis, an iron-dependent modality of regulated cell death, is involved in the immune regulation of cardiovascular disease. However, the relevant regulatory mechanisms of immune infiltration and ferroptosis in VAF have been less studied. In the current study, a highly efficient system for screening immunity- and ferroptosis-related biomarkers and immunomodulatory ability of herbal ingredients has been developed with the integration of intelligent data acquisition, data mining, network pharmacology, and computer-assisted target fishing. VAF patients showed higher infiltration of neutrophils and resting stage dendritic cells, while VSR patients showed higher infiltration of follicular helper T cells. In addition, six (e.g., PCSK2) and 47 (e.g., TGFBR1) ImmDEGs and one (SLC38A1) and four (TGFBR1, HMGB1, CAV1, and CD44) FerDEGs were highly expressed in patients with valvular sinus rhythm (VSR) and VAF, respectively. We further identified a core subnetwork containing 34 hub genes, which were intersected with ImmDEGs and FerDEGs to obtain the key gene TGFBR1. Based on TGFBR1, 14 herbs (e.g., Fructus zizyphi jujubae, Semen Juglandis, and Polygonum cuspidatum) and six herbal ingredients (curcumin, curcumine, D-glucose, hexose, oleovitamin A, and resveratrol) were predicted. Finally, TGFBR1 was found to dock well with curcumin and resveratrol, and it was further verified that curcumin and resveratrol could significantly reduce myocardial fibrosis. We believe that herbs rich in curcumin and resveratrol such as Rhizoma curcumae longae and Curcuma kwangsiensis, mitigate myocardial fibrosis to improve VAF by modulating the TGFβ/Smad signaling pathway. This strategy provides a prospective approach systemically characterizing phenotype-target-herbs relationships based on the tissue-specific biological functions in VAF and brings us new insights into the searching lead compounds from Chinese herbs.

## Graphical Abstract

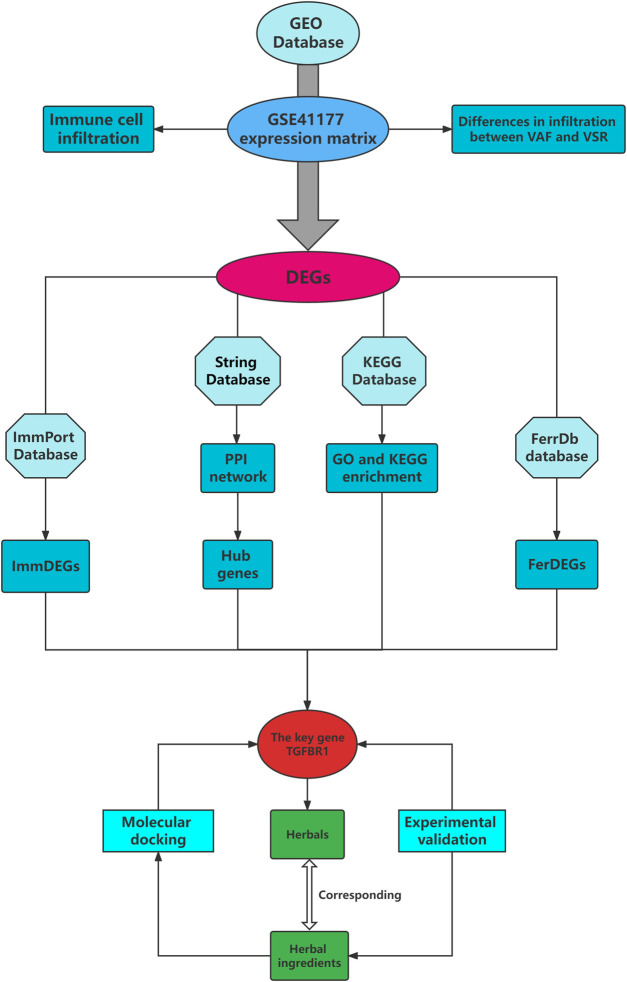

## Introduction

Atrial fibrillation (AF), a common cardiovascular disorder, shows considerably high prevalence across the world, with age being the most important risk factor (Kornej et al., 2020). AF (Table 1) is the most common persistent arrhythmia (Chiang et al., 2013) and an important contributor to stroke, which is the second leading cause of death worldwide (Pistoia et al., 2016). AF has been found to be present in approximately 10% patients with stroke at the time of the attack (Freedman et al., 2017). In fact, considering gaps in monitoring, this percentage is bound to be higher. Haeusler *et al.* on continuous surveillance detected AF in >30% patients with cryptogenic stroke (Haeusler et al., 2018). It is notable that cardiogenic stroke is more severe than other stroke subtypes (Kamel and Healey, 2017). AF is a significant contributor to cardiovascular mortality (Hohendanner et al., 2018), such as myocardial infarction and heart failure (Ruddox et al., 2017; Carlisle et al., 2019). AF and heart failure reportedly co-exist in up to 30% patients owing to numerous shared pathophysiological mechanisms that facilitate the maintenance of each condition (Prabhu et al., 2017). AF can be divided into valvular and non-valvular AF, the former is typically associated with worse prognosis. Valvular atrial fibrillation (VAF) is one of the common clinical manifestations of valvular heart disease (VHD), and VAF can in turn exacerbate VHD (Gaborit et al., 2005). The timing of intervention in asymptomatic patients with VHD remains controversial, interventions are usually initiated when a decline in exercise capacity is observed or when there is shortness of breath (Baumgartner et al., 2020). Consequently, the risk of death always persists when patients develop severe VAF symptoms (e.g., panic palpitations and restricted activity). Anticoagulation therapy is the most basic method to treat VAF, but treatment efficacy becomes limited with disease progress (Lip et al., 2019). Valve replacement is another commonly used treatment method, but complications such as re-thrombosis and recurrent AF pose a challenge. Valvular sinus rhythm (VSR) represents the early stage of VHD, and as the disease progresses, it evolves into VAF, which is one of the most severe stages of VHD. The pathogenesis of AF remains poorly understood, inflammatory signals are apparently one of the determinants of progressive exacerbation of AF (Nattel et al., 2020). The accumulation of immune cells, such as macrophages, in atrial tissue mediates inflammatory responses, resulting in atrial electrophysiology remodeling (Sun et al., 2016). This inflammatory pathological response increases the incidence of AF, and a mutually reinforcing vicious circle is created (Hu et al., 2015). In addition, ferroptosis plays a potential role in AF. Ferroptosis, an iron-dependent modality of regulated cell death, is distinctly different from cell death mechanisms such as apoptosis, necrosis, and autophagy (Li J. et al., 2020; Tang et al., 2021). Ferroptosis and inflammatory responses promote each other (Sun et al., 2020). Inhibition of ferroptosis has been reported to reduce susceptibility to frequent excessive alcohol consumption-induced AF (Dai et al., 2022). However, only few studies have explored inflammatory responses and mechanisms underlying ferroptosis in VAF. Therefore, we aimed to detect differences in immune cell infiltration and immune- and ferroptosis-related gene expression levels in patients with VAF. Our core goal was to determine how to delay VAF progression. Herbal medicines, a natural treasure trove, contain dozens or even hundreds of ingredients; their mechanisms of action often involve multiple pathways and are thus complex. Numerous herbal medicines have been proven to be effective to prevent and treat cardiovascular diseases (e.g., hypertension) in several randomized controlled trials (Hao et al., 2017). With recent advancements in technologies, methods such as high-throughput sequencing have been widely adopted to study the active ingredients of herbal medicines and to identify target genes regulated by them.

**TABLE 1:** Nonstandard Abbreviations and Acronyms.

Full name	Abbreviation
Atrial fibrillation	AF
Valvular atrial fibrillation	VAF
valvular heart disease	VHD
Valvular sinus rhythm	VSR
differentially expressed genes	DEGs
Immune-related DEGs	ImmDEGs
Ferroptosis-related DEGs	FerDEGs
Protein–protein interaction	PPI
Biological process	BP
Cellular component	CC
Molecular function	MF

Herein we used the Gene Expression Omnibus (GEO) database to obtain information pertaining to local gene expression profiles of patients with VAF and VSR and compared differences in immune cell infiltration and immune- and ferroptosis-related gene expression levels, from the data thus collated, we sought to predict effective herbal medicines to treat VAF.

## Materials and methods

### Gene expression profile of patients with VAF and VSR

Gene expression profile of patients with VAF and VSR was obtained by searching the GEO database (Table 2); gene IDs were collected and then converted into gene symbols.

**TABLE 2:** List of all software and websites used in this study.

Name	Entrance
GEO database	https://www.ncbi.nlm.nih.gov/geo/
R soft and main plug-in package	Version: R 4.1.1; Package: limma, clusterprofiler
ImmPort database	https://www.immport.org/home
String databse	https://cn.string-db.org/
Cytoscape	Version: Cytoscape_v3.9.0; Plug-in: Degree
FerrDb database	http://www.zhounan.org/ferrdb/
KEGG Mapper–Color	https://www.kegg.jp/kegg/mapper/color.htmlv
HERB database	http://herb.ac.cn/
PubChem database	https://pubchem.ncbi.nlm.nih.gov/
ChemOffice	Chem3D 19.0
Uniprot database	https://www.uniprot.org/
PDB database	https://www.rcsb.org/
Autodock vina	Autodock vina 1.1.2

### Analyses of immune cell infiltration and differentially expressed genes (DEGs)

The CIBERSORT deconvolution method was used to study immune cell infiltration. The gene expression profiles were normalized and screened for DEGs using the limma R package based on the cutoff criteria of |logFC| ≥ 1 and adjP value ≤0.05.

### Immune-related DEGs (ImmDEGs) and ferroptosis related DEGs (FerDEGs)

In addition to immune cell infiltration analysis, we studied the differential expression of immune-and ferroptosis-related genes in patients with VAF and VSR. Immune- and ferroptosis-related genes were separately identified from the ImmPort and FerrDb databases, respectively; subsequently, they were intersected with DEGs to obtain a list of ImmDEGs and FerDEGs, respectively.

### Protein-protein interaction (PPI), hub genes, and enrichment analyses

We used the STRING database to subject DEGs to PPI analysis and top 30 genes were filtered based on the MCODE plugin of Cytoscape, these genes were considered to be hub genes. DEGs were also subjected Gene ontology (GO) and Kyoto Encyclopedia of Genes and Genomes (KEGG) pathway enrichment analyses using the R package clusterprofiler (cutoff: *p* ≤ 0.05 and q ≤ 0.05). For the enrichment results, in addition to visualizing them as bubble plots, DEGs were tagged in the interested enrichment pathway of by using the color tool of the KEGG database.

### Key genes and herbal medicine prediction

A key gene was defined as a gene that was a ImmDEG, FerDEG, and hub geneenriched in an immune-related pathway. Based on the identified key genes, we reverse predicted target herbal medicines and ingredients using the HERB database.

### Molecular docking for validation

The protein structure of key genes encoded were downloaded from the PDB database and the structure of predicted herbal ingredients required from Pubchem database. Using Autodock vina tools to molecularly dock the key genes with herbal ingredients and the model of lowest binding free energy was regarded as the best bond way.

### Experimental design

The HL-1 cells were used to construct the AF model (Hu et al., 2021). The HL-1 cell line was purchased from Shanghai (TongPai, China), used for *in vitro* research and cultivated in DMEM containing 10% foetal bovine serum (Gibco, MA, United States) and 0.1 mM norepinephrine in a 37 °C cell incubator with 5% CO2. Prior to each experiment, HL-1 cells were inoculated in six-well plates and treated as described below when cells reached 70% confluency. Normal control group (NC): HL-1 cells were cultured in DMEM for 48 h. AngII group (AG): HL-1 cells were incubated with 200 nM AngII for 48 h. Curcumin group (CG): HL-1 was first incubated with curcumin for 2 h in a concentration gradient (0, 5, 25, 50, 100, 250, 400, 1000ug/mL) and then 200 nM AngII was added for 48 h. Resveratrol group (RG): HL-1 was first incubated with Resveratrol for 2 h in a concentration gradient (0, 10, 50, 100, 200, 800, 1600ug/mL) and then 200 nM AngII was added for 48 h.

### Assays of CCK8

The growth status of each group of cells was detected by CCK8 and the effect of each herbal medicine on the viability of HL-1 cells was counted and observed to select the optimal concentration for drug intervention. The method was as follows: approximately 5 × 103 cells were cultiviated in 96 well-plates. Cells were incubated with the CCK8 reagent (10ul) for 2 h at 37°C, follow by observation at an absorbance of 450 nm of light by a Thermomax microplate reader(Molecular devices, CA, United States).

### Detection of qPCR

Total RNA was extracted from cultivated cells by using Trizol regents. cDNA was synthesized by using the EvoM-MLV Kits. RT-PCR was performed using 2X SYBR Green qPCR Master Mix (K1070-500, APExBIO, US) on a CFX96 Real-Time PCR Detection System (Bio-Rad Laboratories) following the manufacturer’s protocol, and analyzed by delta-delta-CT method and given as ratio compared with vehicle control. The following optimized conditions were used: 95 °C for 30 s, 95 °C for 5 s, and 40 cycles at 60 °C for 5 s. The levels of mRNA were normalized in relevance to endogenous GAPDH, and the expression of target genes was analyzed by the method of 2-ΔΔct. The above experiments were repeated three times independently. The primer sequences used in this study are listed in Table 3. All experiments were performed in triplicate.

**TABLE 3:** List of primers for Real-time PCR.

Target	Primer	Sequence (5–3′)
VIMENTIN	FP	CTG​CTT​CAA​GAC​TCG​GTG​GAC
	RP	ATC​TCC​TCC​TCG​TAC​AGG​TCG
Collagen I	FP	AAG​TCA​CCG​AGA​GAA​TTG​TCA​C
	RP	AGA​GAG​CCT​GTC​TTA​GCA​TAT​CC
α-SAM′	FP	GGA​CGT​ACA​ACT​GGT​ATT​GTG​C
	RP	TCG​GCA​GTA​GTC​ACG​AAG​GA

### Statistical analyses

Data were expressed as mean ± standard error of the mean (SEM). Graphpad Prism 9.3.1 software was used to perform unpaired Student’s t-tests to analyse differences in quantitative variables between groups and to construct statistical histograms. *p* value ≤ 0.05 was considered as indicating statistically significant differences.

## Results

### Gene expression profiles

We downloaded the gene expression matrix of GSE41177 (Yeh et al., 2013) (GPL570 platform) from the GEO database. This data matrix contains gene expression level data for patients with VAF(32 samples) and VSR(6 samples), with samples from both the left atria and pulmonary vein and the surrounding left atrial junction.

### Immune cell infiltration and DEGs

**FIGURE 1:**
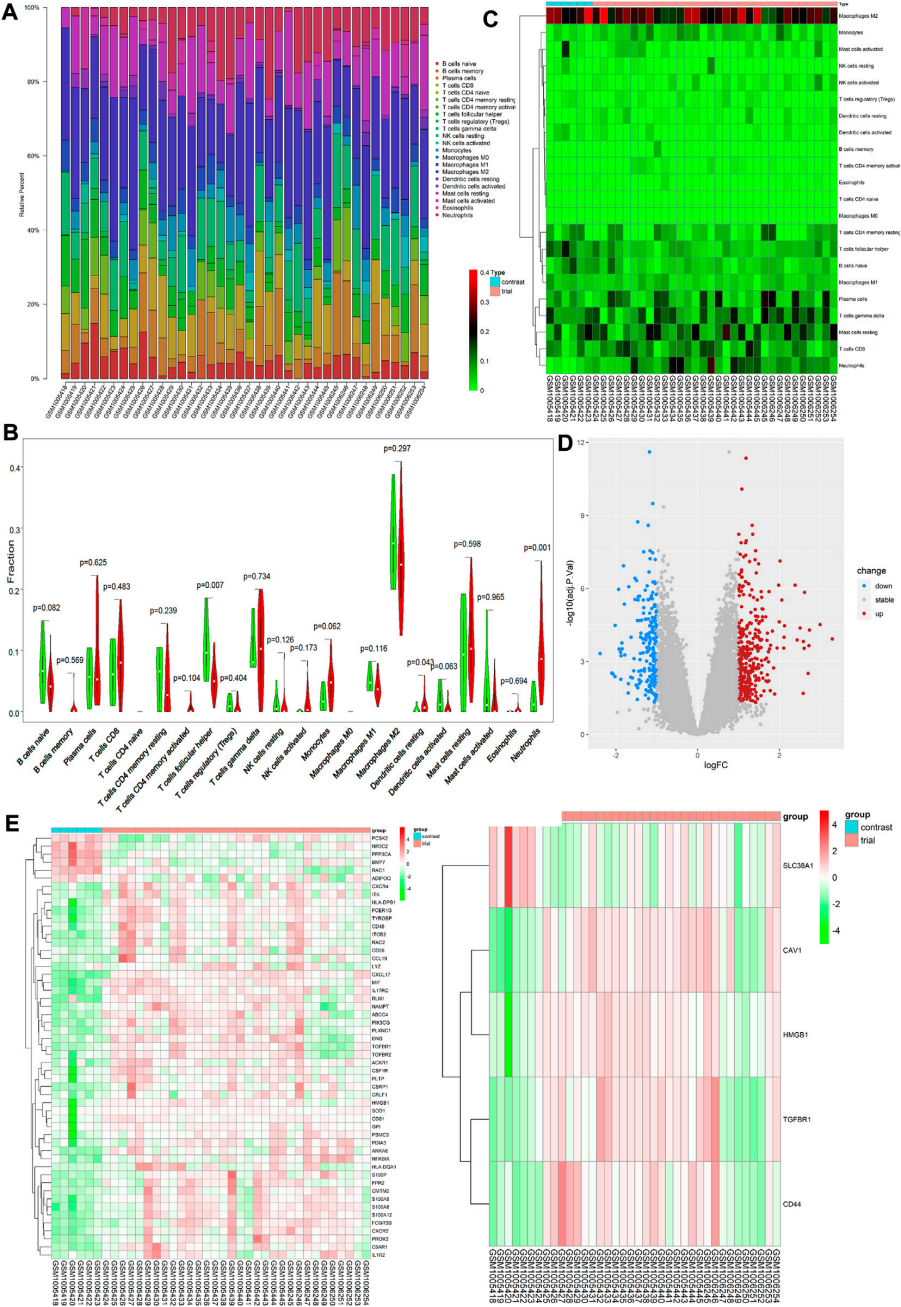
**(A)** Each bar represents a sample, and each color represents a type of immune cell. Area of the color represents the percentage of immune cell infiltration responsible for total immune cell infiltration. **(B)** Each column represents a sample, and each row represents a type of immune cell. Color transition from green to red represents an increase in immune cell infiltration level. **(C)** Red and green violin columns represent patients with VAF and VSR, respectively. The vertical axis represents the ratio of immune cell infiltration responsible for total immune cell infiltration. *p* value, obtained using the Wilcoxon test, represents the difference between the immune cell infiltration level in patients with VSR and VAF. **(D)** Upregulated DEGs are highlighted in red and downregulated DEGs in blue. Criteria: |logFC| ≥ 1 and adjP value ≤0.05. **(E)** Expression levels of 53 ImmDEGs are shown; the darker the red color, the higher the expression level, and the darker the green color, the lower the expression level. **(F)** Expression levels of five FerDEGs are shown; the darker the red color, the higher the expression level, and the darker the green color, the lower the expression level. Contrast group = patients with VSR; trial group = patients with VAF.

On analysis of immune cell infiltration, we initially found differences between patients with VAF and those with VSR (see Figures 1A,C). Subsequently, performing Wilcoxon test, we found that neutrophils (*p* = 0.001) and resting stage dendritic cells (*p* = 0.043) were highly expressed in patients with VAF, while follicular helper T cells (*p* = 0.007) were highly expressed in those with VSR (Figure 1B). We could also identify 585 DEGs: 210 genes were down- and 375 were upregulated (Figure 1D).

### ImmDEGs and FerDEGs

On analyzing immune-related gene expression levels, we identified 53 ImmDEGs: six of them (e.g., PCSK2) were highly expressed in patients with VSR and 47 (e.g., TGFBR1, IL1R2, and CD48) were highly expressed in those with VAF(Figure 1E). Similarly, on analyzing ferroptosis-related gene expression levels, we identified five FerDEGs: one of them, i.e., SLC38A1, was highly expressed in patients with VSR and four (TGFBR1, HMGB1, CAV1, and CD44) were highly expressed in those with VAF (see Figure 1F).

### PPI network construction, hub gene selection, and enrichment analysis

**FIGURE 2:**
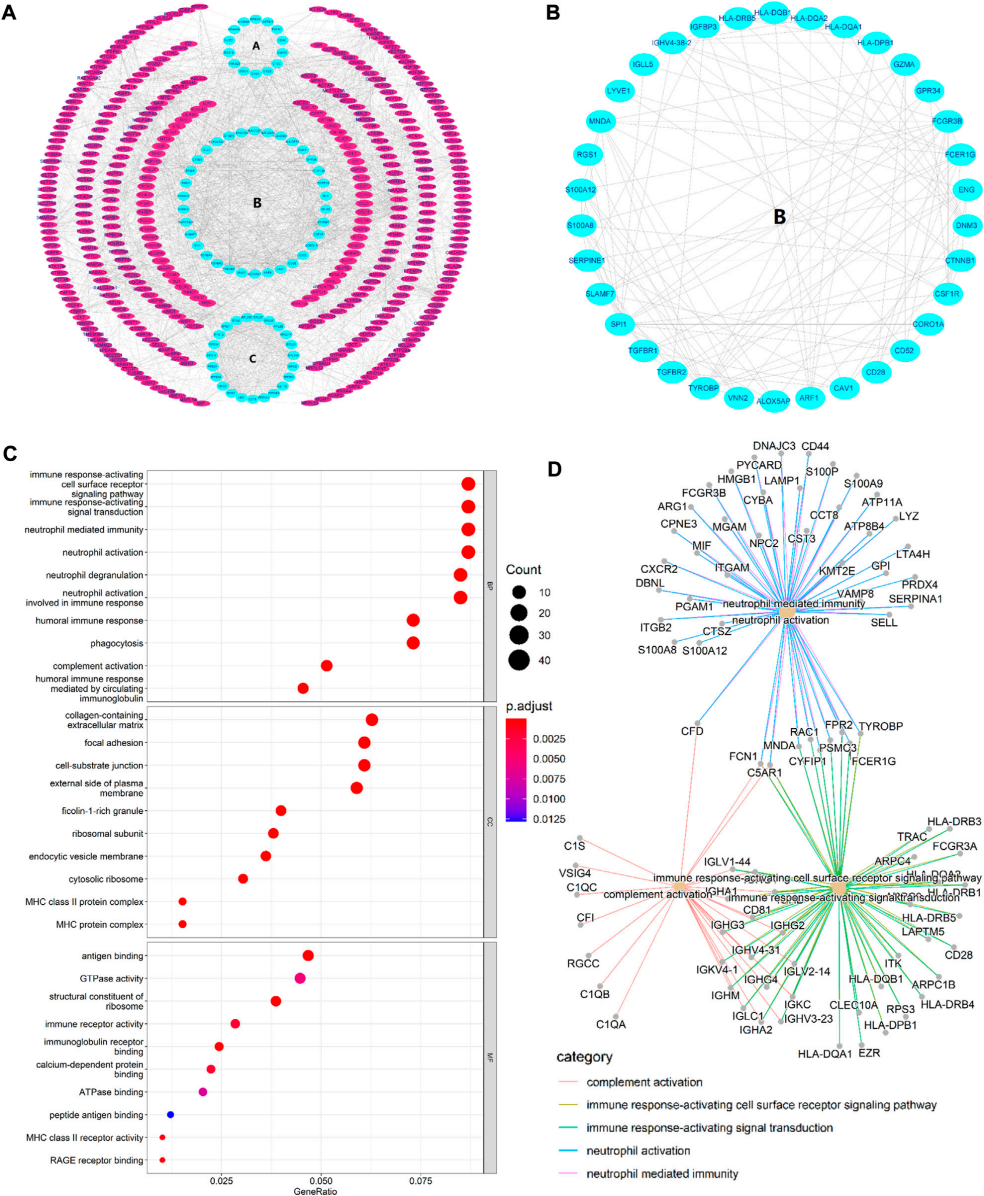
**(A)** PPI network. The three innermost circles represent the top three core subnetworks **(A–C)**. In the network, each node represents a DEG, and the edges represent the correlation between the nodes. In general, the more edges a node had, the greater the role of that node. **(B)** This map shows the core subnetwork **(B)**. Each node represents a hub gene, and the edges represent the correlation between the nodes. In general, the more edges a node had, the greater the role of that node. **(C)** Top 10 GO enrichment results. The horizontal axis represents the gene ratio, i.e., the ratio of the number of DEGs to number of total genes. Dot size is proportional to the gene ratio, and dot color from blue to red that the adjusted *p* value is smaller. **(D)** Association among the top five immune-related biological processes was identified by analyzing co-enriched DEGs.

**FIGURE 3:**
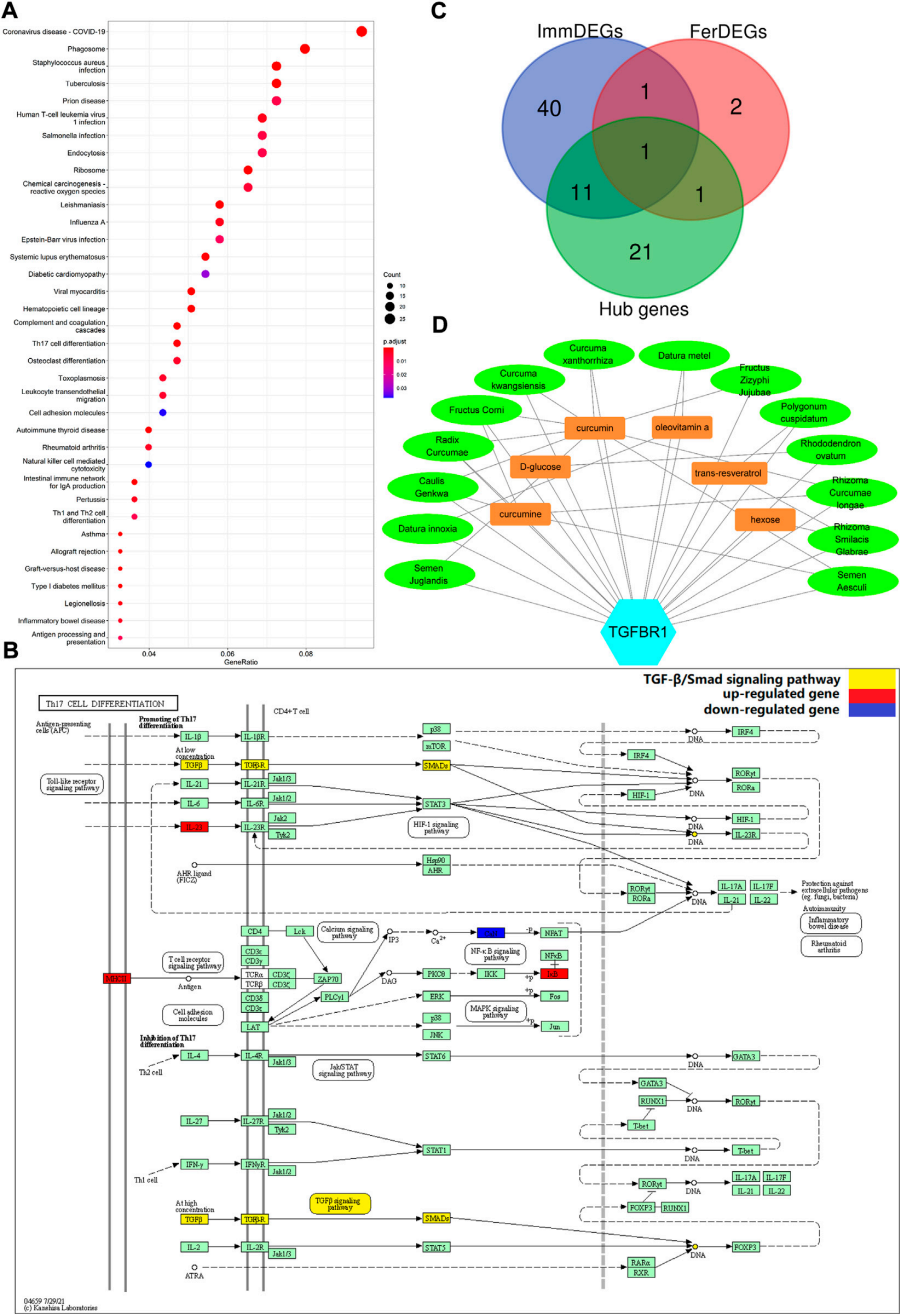
**(A)** Enriched KEGG pathways (n = 36) are shown. The horizontal axis represents the ratio of the number of DEGs enriched in a pathway to the total number of genes in the pathway. Dot size is proportional to the gene-ration, and dot color from blue to red implies that the adjusted *p* value is smaller. **(B)** Th17 cell differentiation pathway. Red nodes represent upregulated DEGs, blue nodes represent downregulated DEGs; the pathway marked in yellow is the TGFβ/Smad signaling pathway. **(C)** TGFBR1 at the intersection of ImmDEGs, FerDEGs, and hub genes. TGFBR1 and CAV1 were the intersecting genes between hub genes and FerDEGs, andCD28, ENG, S100A12, HLA-DPB1, HLA-DQA1, TGFBR1, TGFBR2, TYROBP, CSF1R, FCGR3B, FCER1G, and S100A8 were the intersecting genes between hub genes and ImmDEGs. **(D)** This network displays the correspondence between herbal medicines and herbal ingredients. The blue hexagon represents our key genes, TGFBR1. Brown rectangles represent the six predicted herbal ingredients and green ovals represent the 14 predicted herbal medicines. The lines between the herbal medicines and herbal ingredients show that they have some correspondence.

STRING database was used to perform the PPI analysis of DEGs with the medium confidence ≥0.4 and the top three core subnetworks were screened using the MCODE plugin of Cytoscape (Figure 2A), and hub genes were subsequently selected. The largest core subnetwork, subnetwork B, which contains 34 hub genes (Figure 2B). GO enrichment analysis(see Figure 2C) revealed that DEGs were enriched in 209 biological processes (GO-BP) mainly associated with, for example, immune response-activating cell surface receptor signaling pathway and lymphocyte-mediated immunity. DEGs were also enriched in 62 cellular components (GO-CC), and the major categories represented included, for example, MHC class II protein complex and immunoglobulin complex; similarly, DEGs were enriched in 12 molecular functions(GO-MF), with the major categories being structural constituent of ribosome and immunoglobulin receptor binding. In addition, we explored the connection among immune-related BP by analyzing co-enriched DEGs found that some DEGs, such as FCN1 and FPR2, were co-enriched in multiple BP; they were observed to participate in neutrophil-mediated immunity, immune response-activating cell surface receptor signaling pathway, as well as complement activation (Figure 2D). With regard to KEGG pathway enrichment analysis, 36 pathways, such as Th17 cell differentiation and endocytosis, were enriched (Figure 3A). We chose the TGFβ signaling pathway for further analysis, which is important for Th17 cell differentiation (Figure 3B).

### Key genes and predicted herbal medicines

On intersecting ImmDEGs, FerDEGs, and hub genes, we obtained two common genes: TGFBR1 and HMGB1 (Figure 3C). We selected TGFBR1 as the key gene after comprehensive analyses of pertinent immune-related KEGG pathway, and 14 herbs (*Fructus zizyphi jujubae*, *Curcuma kwangsiensis*, Semen Juglandis, *Polygonum cuspidatum*, *Curcuma xanthorrhiza*, *Rhizoma curcumae longae*, *Rhododendron ovatum*, *Datura metel*, *Datura innoxia*, Fructus Corni, *Semen aesculi*, *Rhizoma Smilacis Glabrae*, *Radix Curcumae*, and *Caulis genkwa*) and six ingredients (curcumin, curcumine, D-glucose, hexose, oleovitamin A, and resveratrol) were consequently predicted (Table 4). There was a correspondence between herbs and herbal medicines ingredients, such as curcumin, in *Rhizoma curcumae longae* and *Curcuma kwangsiensis*, Semen Juglandis and *Radix Curcumae*, amongst others. A visual network diagram (Figure 3D) was constructed to clearly present the relationship between TGFBR1 and herbs/ingredients.

**TABLE 4:** List of 14 herbal medicines and six herbal ingredients predicted in this study.

Herbal ingredients	Herbal medicines		
curcumin	Curcuma kwangsiensis	Caulis Genkwa	Curcuma xanthorrhiza
	Radix Curcumae	Fructus Corni	Semen Aesculi
	Semen Juglandis	Rhizoma Curcumae longae	
curcumine	Rhizoma Curcumae longae	Radix Curcumae	Fructus Corni
	Semen Aesculi	Caulis Genkwa	Semen Juglandis
resveratrol	Polygonum cuspidatum	Rhizoma Smilacis Glabrae	
oleovitamin a	Datura metel	Datura innoxia	
D-glucose	Rhododendron ovatum		
Hexose	Rhizoma Smilacis Glabrae		

### Molecular docking

**FIGURE 4:**
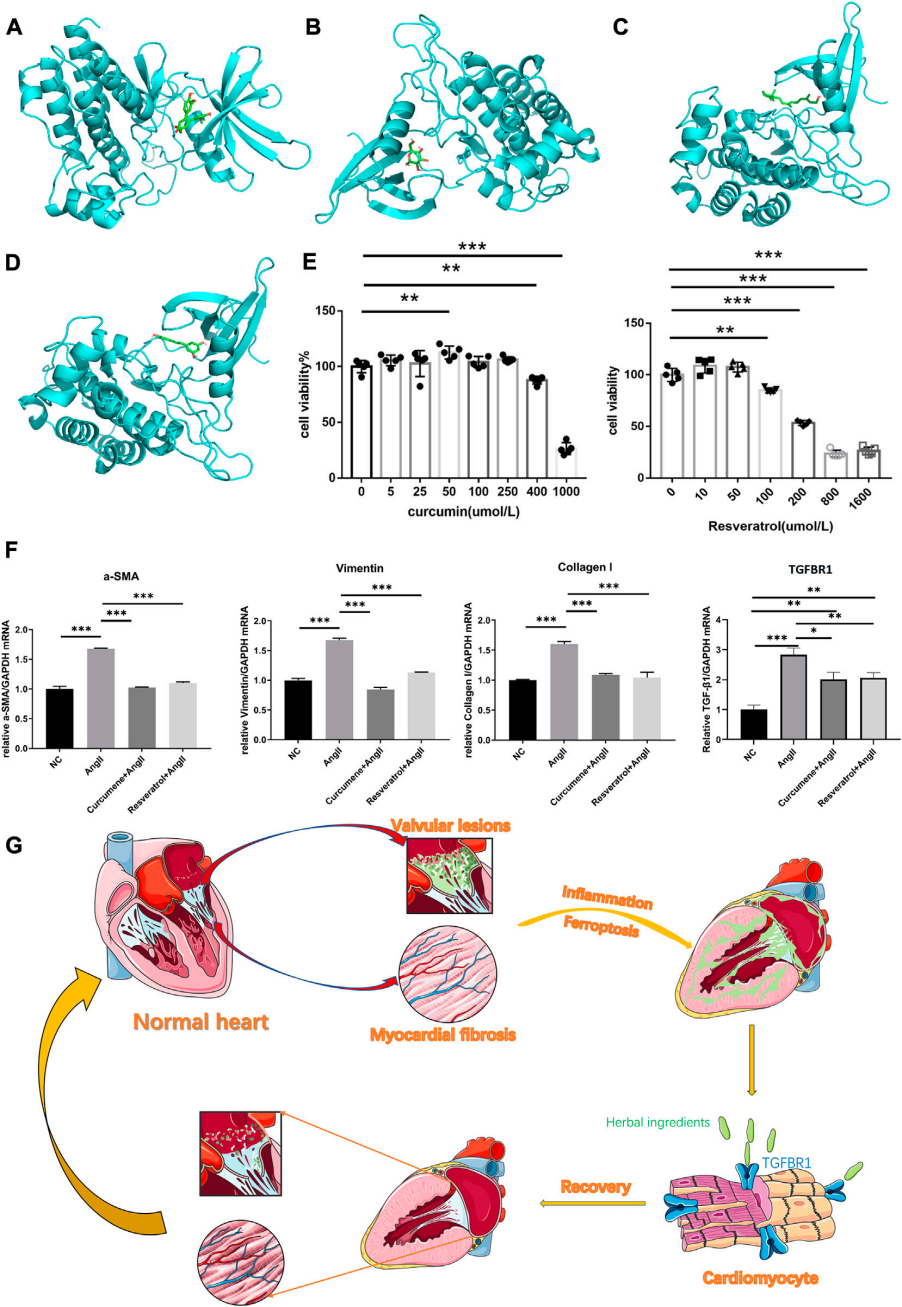
**(A)** The best binding model of curcumin to TGFBR1 with a minimum binding free energy of 8.6 kcal/mol. **(B)** The best binding model of D-glucose to TGFBR1 with a minimum binding free energy of 5.7 kcal/mol. **(C)** The best binding model of oleovitamin A to TGFBR1 with a minimum binding free energy of 7.2 kcal/mol. **(D)** The best binding model of resveratrol to TGFBR1 with a minimum binding free energy of 8.5 kcal/mol **(E)** CCK-8 assays was used to measure the viability of HL-1 cells. **(F)** Quantitative reverse transcription-PCR was used to measure the key gene expression levels. Data are shown as mean ± standard error of the mean. *p < 0.05, **p < 0.01, ***p < 0.001. **(G)** The potential underlying mechanism map.

Our molecular docking results showed that curcumin and resveratrol docked well with TGFBR1, while D-glucose and oily vitamin A did not bind very tightly to TGFBR1(Figures 4A–D). Unfortunately, we were failed to complete the molecular docking of curcumin and hexose to TGFBR1 due to the unavailability of the 2D or 3D structures of curcumine and Hexose. Usually, lower binding free energy results in higher binding model stability. Apparently, it was easy to find that curcumin and resveratrol bound most strongly to TGFBR1, so we selected these two active ingredients as the target components for subsequent CCK8 and qPCR experiments.

### Assays of CCK8

The CCK8 results showed that the intervening concentration of curcumin increased HL-1 cell activity at 50umol/L, did not affect HL-1 cell viability at the remaining low to medium concentrations (≤250umol/L), and significantly inhibited HL-1 activity at high concentrations (≥400umol/L). Resveratrol had no significant effect on HL-1 cell activity at low concentrations (≤50umol/L) and significantly inhibited HL-1 cell activity at high concentrations (≥100umol/L) (see Figure 4E). Therefore, both curcumin and resveratrol were selected at a concentration of 50umol/L in subsequent qPCR experiments.

### Detection of qPCR

At this intervention level, the qPCR results showed that the expression of TGFBR1, vimentin, α-SMA and collagen I was significantly lower in NC, CG and RG than in AG. Surprisingly, vimentin, α-SMA and collagen I in CG and RG were not significantly different from NC. However, although TGFBR1 was significantly lower in CG and RG compared to the AG group, it was still higher compared to NC (Figure 4F).

## Discussion

We herein found that patients with VAF and VSR showed differences in gene expression and immune cell infiltration levels. In comparison to patients with VSR, 375 up- and 210 down-regulated genes were identified in those with VAF. Further, in local cardiac tissue, patients with VAF showed higher infiltration of neutrophils and resting stage dendritic cells, while those with VSR showed higher infiltration of follicular helper T cells. Neutrophil-mediated inflammatory responses are involved in a variety of cardiovascular diseases (e.g., AF, myocardial infarction, heart failure), which is mainly associated with neutrophil extracellular traps (NETs) that recruit other inflammatory cells such as macrophages to amplify the inflammatory response and promotes collagen synthesis in cardiac tissue leading to fibrosis (Warnatsch et al., 2015; Döring et al., 2020; He et al., 2021; Ling and Xu, 2021).

Dendritic cells are most powerful antigen-presenting cells derived from bone marrow and are essential for stimulating adaptive immunity produced by T cells as well as an important bridge between innate and adaptive immunity, and has been found to be associated with cardiac valve tissue inflammation (Skowasch et al., 2005; Waisman et al., 2017; Collin and Bigley, 2018). Dendritic cells in damaged heart tissue can also secrete inflammatory factors and directly activate fibroblasts to proliferate (Lee et al., 2018), a process that promotes the production of collagen fibres. Resting stage Dendritic cells are predominantly found in peripheral tissues and are specifically responsible for antigen capture rather than antigen presentation, which reserving them for the future initiation of T cell-mediated immune responses (van Duivenvoorde et al., 2006; Tiberio et al., 2018). High infiltration of resting Dendritic cells in VAF without activation may indicate that the local tissue immune response is not strong, persistent low levels of inflammation may be an explanation. Unfortunately, Chronic inflammation leads to tissue damage and this damage process is usually accompanied by fibrotic repair, thus creating a vicious cycle of inflammatory damage and fibrotic repair, which eventually leads to continuous cardiac fibrosis that is closely associated with the development of AF (Abe et al., 2018; Smolgovsky et al., 2021). Follicular helper T cells are a specific subset of T cells that are essential for germinal centre formation, differentiation and maturation of B-cell (Choi and Crotty, 2021). This sort of T cells are usually found in inflamed tissues of secondary lymphoid and non-lymphoid organs and provide auxiliary support to B cells such as stimulating them to produce antibodies (Hutloff, 2018; Yoshitomi and Ueno, 2021). It has been reported that such cells are significantly associated with pulmonary fibrosis, skin fibrosis and systemic sclerosis, and that the main mechanism may be related to immune disorders leading to excessive accumulation of antibodies to form inflammatory fibrotic repair after immune damage (Clark, 2018; Beurier et al., 2021; Zhang et al., 2021). However, it is still very poorly studied in cardiac tissue fibrosis, and only very few studies have reported finding that this cell is associated with the cardiac inflammatory response such as in heart transplants, where it enhances the function of B cells to promote a chronic inflammatory response (Wang Y. et al., 2020). Interestingly, in the VSR group, there was a high infiltration of Follicular helper T cells but not B cells, suggesting at least that the accumulation of antibodies formed during the VSR period was not too high, possibly reflecting, to some extent, only mild fibrosis in the heart tissue during this period. Thus, the immune infiltration findings were more prone to suggest chronic inflammation in both VSR and VAF, with post-inflammatory damage followed by fibrotic repair throughout the evolution of VSR to VAF.

Furthermore, we discovered 47 ImmDEGs that were highly expressed in patients with VAF (e.g., TGFBR1, IL1R2, and CD48) and six that were lowly expressed (e.g., PCSK2). Four FerDEGs (TGFBR1, CAV1, HMGB1, and CD44) were also highly expressed in the VAF group, whereas one (SLC38A1) was lowly expressed. Interestingly, TGFBR1 and HMGB1, two intersecting genes between ImmDEGs and FerDEGs, were both highly expressed in the VAF group. The crosstalk between immune response and ferroptosis was well established and it has been investigated for the treatment of tumours such as using activation of CD8^+^ to induce ferroptosis in tumour cells (Tang et al., 2020). The crosstalk between immune cells and ferroptosis can occur in three ways: by the immune cells themselves produce ferroptosis when immune disorder; by tissue cells where ferroptosis is recognised by immune cells and produces an inflammatory clearance response; above both are simultaneously exist (Chen et al., 2021; Yao et al., 2021). In essence, both of way are inflammatory responses, with a sustained inflammatory response leading to further tissue damage and subsequent repair, this repair process that inevitably involves increased secretion and even accumulation of collagen fibres leading to tissue fibrosis. It is clear that this mechanism is likely to be present in the process of evolution from VSR to VAF.

On further analyses, we found that TGFBR1 was the intersecting gene between not only ImmDEGs and FerDEGs but also hub genes. Therefore, we herein considered TGFBR1 as the key gene. TGFBR1, a pleiotropic cytokine, plays a pivotal role in immune response and mediates a vicious cycle of inflammation and tissue fibrosis (Bonniaud et al., 2005; Esebanmen and Langridge, 2017). In fact, TGFBR1 is also involved in tissue fibrosis during ferroptosis. Li et al. reported that ferroptosis inhibitor liproxstatin-1 alleviates radiation-induced lung fibrosis via TGFBR1 downregulation (Li et al., 2019). An increasing body of evidence indicates that AF development is associated with atrial myocardial fibrosis, which presumably underlies the pathology of this persistent arrhythmia (Dzeshka et al., 2015; Jalife and Kaur, 2015; Sohns and Marrouche, 2020). In cardiac tissue, TGFBR1 is evidently involved in the process of tissue fibrosis that can cause VAF and it has been found to cause or exacerbate AF by promoting atrial tissue fibrosis (Khalil et al., 2017; Liu Y. et al., 2021). Wang et al. suggested that quercetin alleviates AF by inhibiting fibrosis of atrial tissues through inhibiting the TGF-β/Smads signaling pathway (Wang et al., 2021). Khalil et al. reported that TGFBR1 participates in tissue fibrosis primarily via the TGFβ-/Smad signaling pathway (Khalil et al., 2017). In traditional Chinese medicine, some herbs, such as Taohong Siwu, have been also observed to significantly attenuate myocardial fibrosis by inhibiting fibrosis proliferation and collagen deposition via this pathway (Tan et al., 2021). In the present study, we also found TGFβ-/Smad signaling pathway to be significantly enriched as a sub-pathway of Th17 cell differentiation. Based on TGFBR1, we predicted six herbal ingredients and 14 herbal medicines. Some of the herbal ingredients identified herein reportedly alleviate tissue fibrosis by modulating the TGF-β/Smad signaling pathway; for example, curcumin has been reported to attenuate pulmonary, hepatic, and renal interstitial fibrosis (Saidi et al., 2019; Wang Z. et al., 2020; Kong et al., 2020). Moreover, curcumin has been found to be effective for treating cardiovascular diseases, such as heart failure, myocardial infarction, atherosclerosis (Li H. et al., 2020), and and it can significantly inhibit the duration of atrial fibrillation episodes, attenuate cardiac fibrosis (Yue et al., 2021). However, there are fewer studies on curcumin’s anti-fibrotic effects through its action on TGFBR1. Therefore, using *in vitro* models and qPCR assays, we found that curcumin significantly reduced the expression of TGFBR1 and fibrosis indicators that Vimentin, α-SMA, collagen I are common indicators of myocardial fibrosis (Ma et al., 2018; Liu M. et al., 2021), which tentatively confirmed the potential of this substance to improve VAF by interfering with TGFBR1 to reduce atrial tissue fibrosis. Curcumin has also been shown to exert anti-inflammatory effects by inhibiting neutrophil infiltration (Antoine et al., 2013), which may also be one of mechanism for reducing myocardial fibrosis.

Resveratrol, another popular herbal ingredient, has also shown good efficacy in the treatment of several cardiovascular diseases (Baczko and Light, 2015; Chong et al., 2015; Yousefian et al., 2019). Resveratrol can effectively improve atrial fibrillation by inhibiting NADPH oxidase and ion channels (Barangi et al., 2018), and like curcumin, it also acts as an anti-inflammatory agent by inhibiting neutrophil activation (Tsai et al., 2019), and attenuates myocardial ischemia-reperfusion injury by inhibiting ferroptosis (Li et al., 2022). However, direct evidence that resveratrol ameliorate atrial myocardial fibrosis is still lacking, so we evaluated their efficacy by intervening in fibrotic HL-1 cells with resveratrol. Same as curcumin, the qPCR results confirmed that resveratrol significantly reduced the expression of TGFBR1 as well as the indicator of fibrosis including Vimentin, α-SMA and collagen I in HL-1 induced by AngII, which given a robust evidence for this potential candidate to act as a treatment for AVF. Therefore, curcumin and resveratrol have great potential to improve AF by acting on TGFBR1 expression to reduce myocardial fibrosis. Oleovitamin A and D-glucose are readily available from daily foods, they have been less studied in AF and VHD, and our molecular docking results suggest that they do not bind very strongly to TGFBR1, and thus we believe they might have less potential to treat VAF.

## Conclusion

We believe that herbs rich in curcumin, resveratrol, such as *Rhizoma curcumae longae*, *Curcuma xanthorrhiza*, and *Caulis genkwa*, attenuate myocardial fibrosis to alleviate VAF by acting on TGFBR1 (see Figure 4G for the potential underlying mechanism), they seem to be effective treatment strategy for VAF.

## Data Availability

The original contributions presented in the study are included in the article/supplementary material further inquiries can be directed to the corresponding author.
